# Health gains and financial risk protection afforded by public financing of selected malaria interventions in Ethiopia: an extended cost-effectiveness analysis

**DOI:** 10.1186/s12936-020-3103-5

**Published:** 2020-01-23

**Authors:** Lelisa Fekadu Assebe, Xiaoxiao Jiang Kwete, Dan Wang, Lingrui Liu, Ole Frithjof Norheim, Abdulrahman Jbaily, Stéphane Verguet, Kjell Arne Johansson, Mieraf Taddesse Tolla

**Affiliations:** 1 Department of Global Public Health and Primary Care Medicine, University of Bergen, Bergen, Norway; 2 Department of Global Health and Population, Harvard T.H. Chan School of Public Health, Harvard University, Boston, MA USA; 3 School of Public Policy and Administration, Xi’an Jiaotong University, Xi’an, Shaanxi China; 4 Department of Health Policy and Management, Yale School of Public Health, New Haven, CT USA; 5 Global Health Leadership Initiative, Yale University, New Haven, CT USA

**Keywords:** Malaria, Ethiopia, Equity, Financial risk protection, Extended cost-effectiveness analysis

## Abstract

**Background:**

Malaria is a public health burden and a major cause for morbidity and mortality in Ethiopia. Malaria also places a substantial financial burden on families and Ethiopia’s national economy. Economic evaluations, with evidence on equity and financial risk protection (FRP), are therefore essential to support decision-making for policymakers to identify best buys amongst possible malaria interventions. The aim of this study is to estimate the expected health and FRP benefits of universal public financing of key malaria interventions in Ethiopia.

**Methods:**

Using extended cost-effectiveness analysis (ECEA), the potential health and FRP benefits were estimated, and their distributions across socio-economic groups, of publicly financing a 10% coverage increase in artemisinin-based combination therapy (ACT), long-lasting insecticide-treated bed nets (LLIN), indoor residual spraying (IRS), and malaria vaccine (hypothetical).

**Results:**

ACT, LLIN, IRS, and vaccine would avert 358, 188, 107 and 38 deaths, respectively, each year at a net government cost of $5.7, 16.5, 32.6, and 5.1 million, respectively. The annual cost of implementing IRS would be two times higher than that of the LLIN interventions, and would be the main driver of the total costs. The averted deaths would be mainly concentrated in the poorest two income quintiles. The four interventions would eliminate about $4,627,800 of private health expenditures, and the poorest income quintiles would see the greatest FRP benefits. ACT and LLINs would have the largest impact on malaria-related deaths averted and FRP benefits.

**Conclusions:**

ACT, LLIN, IRS, and vaccine interventions would bring large health and financial benefits to the poorest households in Ethiopia.

## Background

Malaria prevention and control has been prioritized over the past decade in many national health sector plans. As a result, remarkable progress was made worldwide in reducing incidence and mortality from malaria [1, 2]. Due to the expansion of effective strategies, between 2001 and 2013, malaria incidence has dropped by 30% [1, 2]. Despite such progress, malaria remains a major public health burden with a huge impact on the socio-economic development of many countries [1, 2]. Nearly one-half of the world population lives in malaria-endemic countries [3]. In 2016 alone, there were an estimated 216 million cases and 445,000 deaths attributable to malaria worldwide [4]. Sub-Saharan Africa accounts for 90% of both cases and deaths due to malaria [4]. Malaria control is unequally distributed across socioeconomic groups and the rates of insecticide- and drug-resistance are increasing. Further scale-up of cost-effective malaria interventions with sustainable financing mechanisms is therefore urgently needed [5].

Ethiopia has made notable progress towards malaria control [6, 7]. Nationally, the prevalence of malaria has declined from 5 to 3% over 2010–2015 [5, 8, 9]. During the same period, malaria-related deaths were reduced by 40% [5]. Scale-up of effective anti-malaria interventions at the primary health care level and improved community engagement were major contributing factors to this progress [10]. There is little evidence from Ethiopia about other factors that might have contributed to malaria decline (e.g. climate change, housing structures and urbanization). However, despite significant progress, much remains to be done in the fight against malaria in Ethiopia, where about 2.6 million cases and 5000 deaths were estimated for the year 2016 [4]. Additionally, the 2015 malaria indicator survey shows that only 40% of the population at risk correctly use insecticide-treated bed nets [9].

Malaria prevention and control are major priorities for Ethiopia’s health sector transformation plan (HSTP) [11]. The primary strategies include rolling out long-lasting insecticide-treated bed nets (LLIN) and insecticide residual spray (IRS) for at-risk population [10, 12]. Similarly, artemisinin-based combination therapy (ACT) is recommended as first-line treatment of uncomplicated malaria [10, 12]. Ethiopia has committed to end malaria by 2030 and adopted global malaria control and elimination strategies [12]. As the country moves towards elimination by 2030, tests that are more sensitive will be required to detect subclinical malaria infection to prevent disease transmission [13]. A malaria vaccine (i.e. RTS,S/AS01) could help curb the malaria burden. However, the efficacy of the vaccine is partial and presents rapid waning immunity [14, 15].

Malaria is endemic in many regions of Ethiopia with marked seasonal and geographic variation. Nearly 60% of the total population reside in high-risk areas [10, 12]. In addition to its public health impact, malaria imposes a large financial burden on households, consuming on average 7% of household income [16, 17]. Marginalized and economically vulnerable populations are also at a higher risk of acquiring malaria and of experiencing fatal consequences because of limited health care access and the inability to pay for it [1, 18, 19]. Malaria spending is estimated to cost Ethiopia about $200 million annually or 10% of its total health expenditure [20]. Hence, reducing malaria disease burden has the potential to improve socioeconomic development [21].

The recent attention to universal health coverage (UHC) has provided context to explore mechanisms that would expand access to malaria prevention and treatment services in Ethiopia [22]. This would also help address the high rate (33%) of out-of-pocket (OOP) payments [20]. Given that a quarter of the Ethiopian population lives below the national poverty line [23], OOP malaria treatment costs can be an important barrier to access effective treatment and in pushing households into impoverishment in Ethiopia. Accounting for non-health benefits is essential to reduce health inequalities and contribute to the objectives of UHC [22]. Financial risk protection (FRP) is an important policy objective and can improve access to all needed quality health services without financial hardship [24, 25].

In this paper, the aim is to estimate the potential health, FRP, and equity benefits of universal public finance of scaling up selected malaria prevention and treatment interventions in Ethiopia [26]. This will support policymakers in jointly considering health gains, FRP and equity benefits in resource allocation related decisions.

## Methods

Using extended cost-effectiveness analysis (ECEA), we consider the costs and health impact of malaria interventions across population subgroups and estimate the FRP impact on households in Ethiopia [26]. Building on a recent ECEA of malaria vaccine [28], and using a static disease model, are quantified, across socioeconomic groups (i.e. income quintiles), for each of four malaria interventions (ACT, LLIN, IRS, and malaria vaccine): the number of malaria-related deaths and OOP expenditures averted; the corresponding household FRP provided; and the implementation costs. Furthermore, ECEA is also applied across malaria transmission intensities to account for geographic variation of malaria (see Additional file 1: Appendix Table S2).

### Malaria interventions

Large scale use of LLINs is a key strategy to reduce malaria burden [29]. A meta-analysis showed that LLIN was effective in both reducing malaria cases (by 50%) and malaria deaths (by 18%) [27]. IRS can eliminate malaria vectors by applying a residual insecticide to the internal walls and ceilings of homes [2, 30], and its use has been shown to decrease plasmodium falciparum malaria by 29% [31]. A complete cure can be expected in 95% of falciparum malaria cases treated with ACT [32]. The proportion of *Plasmodium falciparum* malaria in Ethiopia totals about 80–90% of all malaria cases [9]. Lastly, a recent clinical trial showed a 26% reduction in the number of episodes and hospital admissions, in children under 2 years of age, following three doses of malaria vaccine (currently under development) [14].

### Health benefits

Population at risk of malaria (accounting for 60% of total population—defined as areas with annual incidence > 0 per 1000 population) is the target population for LLIN and IRS (Table 1) [12]. Similarly, the estimated number of annual malaria cases and birth cohorts born in at-risk areas were the target populations for ACT and vaccine, respectively [12, 14]. Target populations were split into income quintiles for LLIN, IRS, and ACT interventions. As for the vaccine, quintile-specific total fertility rates were applied in order to differentiate between the number of susceptible individuals per income quintile (see Additional file 1: Appendix). For each intervention, in order to calculate malaria prevalence by at-risk population per income quintile, first the relative risk of malaria prevalence by income quintile is estimated for the general population [9, 10]. These stratified relative risks were multiplied by average malaria prevalence, in order to split prevalence rates across income quintiles for populations at risk (see Additional file 1: Appendix) [9, 10].

The baseline coverage (before introduction of universal public financing) was 40% for LLIN and 29% for IRS and their respective coverage by income quintile was sourced from the 2016 malaria indicator survey (MIS) (Table 1) [9]. LLIN use, rather than its possession, was selected as a proxy parameter because the actual use of LLIN reflects behavioural change [33]. The percentage for whom care was sought among children who had fever in the past 2 weeks was used as a proxy for probability of seeking malaria care and baseline ACT coverage (35%) [34, 35]. A 10% incremental coverage across quintiles was assumed for each intervention. For the vaccine, in addition to the 10% incremental increase in coverage, a scenario with coverage scale-up from 0 to 33% was also considered (since this is the national coverage level of the basic child immunization programme) [34].

Before intervention, 2.6 million cases and 5000 deaths attributed to malaria were assumed to occur annually in Ethiopia [4]. On average, 1% of all malaria cases would be hospitalized, according to the integrated disease surveillance database [36, 37]. Severe and mild cases were treated as inpatient and outpatient cases, respectively. Deaths averted by each intervention were calculated as a product of disease incidence, case fatality ratio, intervention efficacy and incremental coverage (see Additional file 1: Appendix).

**Table 1:** Extended cost-effectiveness analysis input parameters for public financing of selected malaria prevention and treatment interventions in Ethiopia

Parameter	Value		References
Epidemiology			
Population at risk of malaria (2016)	61,504,000		[12, 38]
Population for malaria vaccine (2016 birth cohort)	1,984,000		Authors’ calculation [34, 38]
Crude birth and child mortality rate, per 1000 population	32, 20		[34]
Total fertility rate, Q_1_–Q_5_; A^a^	6.4, 5.6, 4.9, 4.3, 2.6; 4.6		[34]
Average household size	4.2		[38]
Number of malaria deaths in the general population, population at risk, and children	5000; 3767; 1790		[4, 39]
Prevalence of malaria in population at risk, Q_1_–Q_5_; A	4.6; 3.1; 3.6; 2.2; 2.1; 3.1%		[9, 10]
Prevalence of malaria in children, Q_1_–Q_5_; A	5, 3.3, 2.9, 2, 1.7, 3.1%		[9]
Probability of seeking malaria care, Q_1_–Q_5_; A	23.8, 30.4, 33.0, 42.3, 50.5; 35.3%		[34]
Case fatality ratio for malaria outpatient and inpatient cases	0.19; 0.65%		[3, 4]
Proportion of malaria-related hospital admissions, Q1–Q5	1.00, 0.90, 0.96, 0.87, 0.83; 0.91%		[36, 37]
Effectiveness of LLIN	50%		[27, 40]
Effectiveness of indoor residual spraying (IRS)	29%		[31]
Vaccine efficacy, Weibull decay after 9 months over 5-years	9–12 months	77%	Authors’ calculation based on [41]
	12–24 months	46%	
	24–36 months	23%	
	36–48 months	13%	
	48–60 months	8%	
Effectiveness of artemisinin combination therapy (ACT) on mortality reduction	95%		[32]
Interventions			
LLIN coverage before intervention, Q_1_–Q_5_, A	26, 36, 42, 47, 44; 40%		[9]
LLIN coverage after intervention, Q_1_–Q_5_, A	36, 46, 52, 57, 54; 50%		[12] Authors’ assumption
IRS coverage before intervention, Q_1_–Q_5_, A	35, 35, 36, 28, 11; 29%		[9]
IRS coverage after intervention, Q_1_–Q_5_, A	45, 45, 46, 38, 21; 39%		[12] Authors’ assumption
Malaria vaccine coverage before intervention, Q_1_–Q_5_, A	0		[15]
Malaria vaccine coverage after intervention, Q_1_–Q_5_, A	10, 10, 10, 10, 10; 10%		Authors’ assumption
Malaria vaccine coverage after intervention, Q_1_–Q_5_, A (fully immunized coverage)	19, 31, 30, 40, 58; 33%		[34]
ACT coverage before intervention, Q_1_–Q_5_, A	24, 30, 33, 42, 51; 35%		[34]
ACT coverage after intervention, Q_1_–Q_5_, A	34, 40, 43, 52, 61; 45%		Authors’ assumption
Costs (2016 $)			
Out-of-pocket outpatient costs, Q_1_–Q_5_, A	$6.4, 6.8, 5.5, 6.6, 5.7; 6.2		[42]
Out-of-pocket inpatient costs	$65.9		[18]
Unit cost of malaria treatment outpatient visit	$7.3		[43]
Unit cost of malaria treatment inpatient visit^b^	$31.6		[43]
Unit cost of LLIN	$5.4		[44]
Unit cost per vaccinated child (3 doses)	$26.0		[45]
IRS unit cost per person protected	$5.3		[46, 47]
Household consumption expenditure Q_1_–Q_5_, A	$227, 369, 499, 671, 1422; 638		[48]
Share of food in total consumption expenditure Q_1_–Q_5_, A	48, 54, 51, 51, 58, 54%		[23]
GDP per capita 2016	$713		[38]

^a^Q_1_ stands for poorest income quintile, Q_5_ for richest income quintile, and A for average

^b^Average unit cost estimate for inpatient visit

### Financial consequences for households

Both inpatient and outpatient care of malaria can impose an economic burden to individual households. Direct medical, non-medical, and indirect costs were extracted from two previously published studies [18, 42]. Before universal public finance (UPF) of each intervention, individuals seeking malaria care would pay about $6 and $66 out-of-pocket (OOP) costs for outpatient and inpatient treatment, respectively [18, 42]. Even if there were no OOP payments for preventive interventions, the three malaria preventive interventions (i.e. LLIN, IRS, vaccine) would lower the risk of malaria and thus household OOP expenditures related to malaria treatment. The amount of OOP expenditures averted per income quintile was quantified, before and after UPF. OOP expenditures averted depended on: target population, incremental coverage, health care use, OOP payments, and preventive intervention effectiveness (see Additional file 1: Appendix).

### Financial risk protection benefits

The financial risk faced by households depends on the malaria burden, intervention coverage, and probability of seeking treatment. Annual consumption expenditures were extracted from the Ethiopian Household Income Consumption and Expenditure and Welfare Monitoring Survey as a proxy for income [48]. In this study, a case of catastrophic health expenditures (CHE) was counted when total OOP spending for malaria treatment exceeded 10% of total household consumption expenditures or 40% of capacity to pay (i.e. non-food total household consumption) [49, 50]. UPF introduction would avert a number of CHE cases following the reduction in incidence of OOP expenditures.

### Intervention costs

The cost of each intervention was estimated from the health system perspective. Average unit cost estimates for preventive (LLIN, IRS, and vaccine) and curative (ACT) interventions were obtained from published studies (Table 1) [44–47]. The unit cost for LLIN included net price and delivery cost. Similarly, for IRS, insecticide cost accounted for 50%, spray campaign operations and labour for 26%, capital cost for 23% and other commodities accounted for 1% [44, 46, 47]. The average unit cost per fully vaccinated child included vaccine price, and supplies accounted for 84%, and the remaining costs (16%) included training, transportation, waste management [45]. Unit cost of ACT comprised of human resources at 58%, drug and pharmaceutical supplies at 25% and rest was indirect costs [43]. Patient and health system costs were extracted from the literature and converted for the year 2016 using Ethiopia’s gross domestic product (GDP) deflator [38]. The total costs considered: target population, intervention coverage and intervention unit cost.

### Sensitivity analyses

The robustness of the findings were tested by using one-way sensitivity analyses. Specifically, the value of malaria prevalence, case fatality ratio, intervention effectiveness, health services utilization, and intervention unit cost were varied by ± 20%, one at a time, to evaluate the interventions impact on the deaths and CHE averted, across income quintiles.

## Results

### Deaths and cases of CHE averted by malaria interventions

Increasing coverage (by 10%) of ACT, LLIN, IRS and vaccine among the population at risk would avert 358, 188, 107 and 38 deaths per year in Ethiopia, respectively. The four interventions would also avert 440 (i.e. 10% of the baseline CHE), 220 (5%), 125 (3%) and 18 (2%) CHE cases annually, respectively. Among the interventions, LLIN and ACT would have the largest number of deaths averted and CHE cases averted. In addition, ACT and LLIN would avert $4,277,000 and $214,000 of OOP expenditure, respectively (Table 2).

**Table 2:** Total government costs, household out-of-pocket (OOP) expenditures averted, deaths averted, and catastrophic health expenditure (CHE) cases averted from universal public finance of selected malaria interventions at 10% incremental coverage, in Ethiopia

Interventions	Net government costs (2016 USD) (incremental)	OOP expenditures averted (2016 USD)	Deaths averted	Cases of CHE averted
Artemisinin-based combination	5,721,000	4,277,000	358	440
Long-lasting insecticide-treated bed nets	16,489,000	214,000	188	220
Indoor residual spray	32,644,600	122,000	107	125
Malaria vaccine	5,144,000	15,000	38	18

### Distribution of deaths and CHE cases averted by malaria intervention

**Fig. 1:**
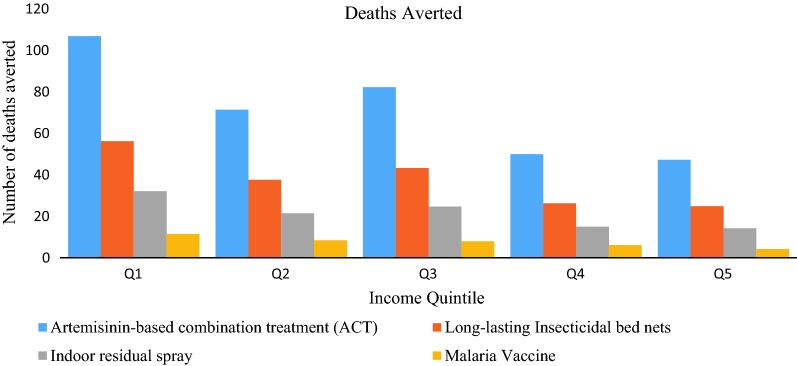
Distribution of deaths averted by each malaria intervention per income quintile in Ethiopia

All four interventions would save larger numbers of lives among the poor, due to the fact that the poor would face a higher malaria prevalence and associated risk factors. For example, ACT would avert twice as many deaths in the poorest income quintile as compared to the richest quintile (Fig. 1). 50% of the deaths averted would be concentrated in the poorest two quintiles. The distribution of deaths averted (by LLIN, IRS and ACT), from poorest to richest quintiles, would be 30, 20, 23, 14 and 13%, respectively. Similarly, the distribution of deaths averted by the malaria vaccine would be 30, 22, 21, 16, and 11%, respectively (Fig. 1).

**Fig. 2:**
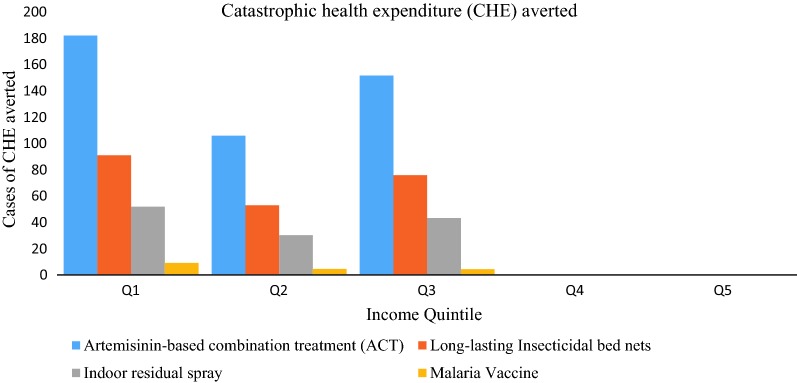
Distribution of financial risk protection benefits (cases of CHE averted at 10% threshold) for each intervention per income quintile in Ethiopia

For each intervention, the gradient in private OOP expenditures averted would be flat across quintiles as malaria prevalence would decrease with increasing income, but the probability of seeking malaria care would increase as income goes up (Table 3). Therefore, the gains in private expenditures would be evenly distributed across income quintiles. Across the first three income quintiles, a greater number of CHE cases would be averted and the largest benefits would be among the poorest income quintile (Fig. 2).

**Table 3:** Out-of-pocket private expenditures averted (in 2016 USD) per income quintile for all malaria interventions in Ethiopia

Interventions	Income group				
	Q1	Q2	Q3	Q4	Q5
Artemisinin-based combination	966,209	847,472	891,970	789,078	782,701
Long-lasting insecticide-treated bed nets	48,310	42,374	44,598	39,454	39,135
Indoor residual spray	27,537	24,153	25,421	22,489	22,307
Malaria vaccine	4879	3659	2556	2278	1215

Q1; poorest quintile, Q5; richest quintile

The annual policy costs of UPF for 10% incremental coverage of ACT, LLIN, IRS and vaccine would be $5.7, 16.5, 32.6, and 5.1 million, respectively. Similarly, due to declines in malaria cases through preventive interventions, $241,000, $137,000 and $16,000 of government expenditures on malaria treatment would be averted annually by LLIN, IRS and malaria vaccine, respectively.

Most of these government savings would be observed within quintile one to three and LLINs would contribute to more than half of these savings. The rollout of malaria vaccines at 10% incremental coverage, under the routine immunization program in the country, would cost around $5 million and avert 38 deaths and reach $17 million and avert about 120 deaths with 33% coverage.

### Deaths and cases of CHE averted per million spent

The health benefits per $1 million invested on ACT, LLIN, IRS, and vaccine interventions would be 63, 11, 3, and 7 lives, respectively. Similarly, they would reduce OOP expenditures by $1,560,000, 13,000, 3700 and 2800, respectively; with varying numbers of CHE cases averted by income quintile (see Additional file 1: Appendix, Figs. S1–S3).

### Sensitivity analyses

The results of our univariate sensitivity analyses are described in Table 4 (and Additional file 1: Tables S3–S6). Generally, the distribution of health gains is highly prone to variations in malaria prevalence, case fatality ratio and intervention efficacy. The distributions in OOP expenditures averted and CHE cases averted would be more sensitive to malaria prevalence, health care utilization, probability of seeking inpatient care, intervention efficacy and OOP expenditures.

**Table 4:** Sensitivity analyses on the impact on deaths and catastrophic health expenditure (CHE) cases averted when long-lasting insecticide-treated bed nets (LLIN) input parameters vary across income quintiles (Q1 = poorest; Q5 = richest), (low to high shows when input parameters are decreased or increased by 20%, respectively)

Sensitivity analysis LLIN	Q1		Q2		Q3		Q4		Q5	
	Low	High	Low	High	Low	High	Low	High	Low	High
Prevalence of malaria										
Deaths averted	45	68	30	45	35	53	21	32	20	30
Private expenditures averted	38,710	58,070	33,900	58,070	36,090	50,850	31,830	54,130	31,810	47,720
Cases of CHE averted	73	109	42	64	61	92	0	0	0	0
Malaria case fatality ratio										
Deaths averted	46	67	30	45	35	52	21	31	20	30
Private expenditures averted	48,310	48,310	42,370	42,370	44,600	44,600	39,450	39,450	39,135	39,135
Cases of CHE averted	91	91	53	53	76	76	0	0	0	0
Health services utilization										
Deaths averted	56	56	38	38	43	43	26	26	25	25
Private expenditures averted	38,970	58,460	33,450	50,180	35,680	53,520	31,340	47,010	31,620	47,430
Cases of CHE averted	73	110	42	63	61	91	0	0	0	0
Probability of inpatient visit										
Deaths averted	56	57	37	38	43	44	26	26	25	25
Private expenditures averted	47,230	49,390	41,750	43,000	43,680	45,500	39,020	39,890	38,660	39,610
Cases of CHE averted	73	109	42	64	61	91	0	0	0	0
Efficacy										
Deaths averted	45	68	30	45	35	52	21	32	20	30
Private expenditures averted	38,650	57,970	33,900	50,850	35,680	53,520	31,560	47,350	31,310	46,960
Cases of CHE averted	73	109	42	64	61	91	0	0	0	0
Cost inputs										
Government costs	2,625,170	3,963,500	2,632,890	3,971,210	2,621,860	3,960,190	2,634,170	3,972,500	2,628,660	3,966,990
OOP outpatient costs										
Deaths averted	56	56	38	38	43	43	26	26	25	25
Private expenditures averted	39,850	56,770	34,600	50,150	36,680	52,520	32,040	46,860	31,820	46,450
Cases of CHE averted	91	91	53	53	76	76	0	0	0	0

## Discussion

In this paper, the health and financial benefits of UPF for malaria interventions were estimated across Ethiopian households at all income levels. Overall, all four interventions showed substantial benefits, with ACT and LLIN accounting for the larger shares of malaria-related deaths and CHE cases averted.

All the interventions showed a greater number of deaths averted among the poorest 40% of the population, averted similar OOP expenditures across all income groups, and relatively higher FRP benefits for the poorest 40%. Even if the poor had lower access for care and higher baseline malaria risk, for each of the intervention greater benefits would go toward the poor. This suggests that the malaria interventions analysed in this paper benefit the worse-off and poor populations in remote areas of Ethiopia, who suffer the disease risk at most. Given the relatively lower malaria burden, the four malaria interventions would avert fewer deaths annually, as compared to, other interventions addressing childhood diarrhoea and pneumonia for example [51, 52]. Rapid decline of malaria deaths in Ethiopia over the last two decades and a relatively lower prevalence were the main reasons [6]. Among the four interventions, LLIN and ACT were the two strategies with the highest impact on malaria mortality. In contrast, the malaria vaccine would prevent the smallest number of deaths averted (i.e. 38 per year) as compared to the other interventions. This is largely because the vaccine would be relatively less efficacious [14, 41]: only 2% of malaria-related child deaths would be prevented from the vaccine in this study.

Even though the rich had more access to health services and less malaria burden, the private OOP savings would be similar across all income quintiles. This might be due to the fact that the poor and rich are spending similar OOP expenditures for malaria care. In absolute terms, the gains in private OOP expenditures could be lower as compared to findings from other Ethiopian ECEAs [51–53]. This might be due to less OOP payments for malaria care as compared to the other diseases. As for the FRP benefits, LLIN and ACT prevented a higher number of CHE cases, and for all interventions, the greatest number of CHE cases averted would occur in the poorest income quintile. In addition, the annual cost of implementing IRS at a 10% incremental coverage for the at-risk population was about $33 million, 2 times higher than that of the LLIN intervention. This corresponds to more than 16% of malaria-related health care spending in Ethiopia [20]. Lastly, though ACT, LLIN, IRS, and malaria vaccine are critical for malaria control and elimination, these interventions would need to be combined with other interventions, such as behavioural change, correct use and implementation, to yield full impact.

Nevertheless, the analysis presented here has several limitations. First, the disease model was static and did not address the dynamics of malaria transmission. Second, because of the unavailability of key input parameters by socioeconomic group, proxy input parameters were used. For example, the percentage who sought treatment for fever in the past 2 weeks was used as a proxy indicator for seeking malaria care. This might have overestimated malaria cases as there are other causes of fever among individuals (besides malaria). The Ethiopian 2016 DHS, the Malaria indicator survey and the ACT malaria consortium guidance on health equity analysis use health care utilisation due to fever in the past 2 weeks as a proxy for seeking care for malaria [9, 34, 35]. Third, due to the lack of disaggregated data, constant rates for case fatality ratio, intervention effectiveness, and inpatient cost inputs were assumed across quintiles. Fourth, unit costs for the vaccine were not specific to Ethiopia. However, despite the limitations, the analysis is crucial as the findings could assist policymakers decide on which health interventions to rollout to reduce malaria disease burden affecting 60% of the Ethiopian population [9].

The ECEA can also answer some of the equity concerns by providing valuable information on how malaria prevention or treatment strategies would decrease both malaria burden and financial risk incurred by households across various socioeconomic groups in Ethiopia. This study shows that malaria interventions could improve FRP across all income groups, especially among the bottom income groups in Ethiopia. Furthermore, this analysis can help reorienting malaria interventions to target elimination across selected segments of the population, especially among the poor.

## Conclusions

All four malaria interventions would save more lives among the poor than among the rich. Preventing and treating malaria provides substantial health benefits and FRP, especially among poor Ethiopians. ACT and LLINs would generate the largest impact on malaria-related deaths averted and FRP benefits. Improving health equity and reducing poverty are major objectives of the Sustainable Development Goals, and the findings of the study presented here would provide insight for policymakers on how to prioritize malaria interventions for targeted population groups including the poorest.

## Supplementary information

**Additional file 1.** Additional Appendix, Figures S1–S3 and Tables S1–S6.

## Data Availability

Not applicable.

## Abbreviations

ACT: artemisinin-based combination therapy
CFR: case fatality ratio
CHE: catastrophic health expenditure
CTP: capacity to pay
ECEA: extended cost-effectiveness analysis
EDHS: Ethiopia Demographic and Health Survey
FRP: financial risk protection
GDP: gross domestic product
IRS: indoor residual spraying
LLIN: long-lasting insecticidal nets
MIS: malaria indicator survey
OOP: out-of-pocket payment
UHC: universal health coverage
UPF: universal public financing
USD: United States dollar
WHO: World Health Organization
