# Supplementary material for: Health gains and financial risk protection afforded by public financing of selected malaria interventions in Ethiopia: an extended cost-effectiveness analysis
Source: Malar J. 2020 Jan 23;19:41. doi: 10.1186/s12936-020-3103-5 (PMC6979328; doi:10.1186/s12936-020-3103-5)
Supplement: Supplementary file 1 — Additional file 1. Additional Appendix, Figures S1–S3 and Tables S1–S6. [file 12936_2020_3103_MOESM1_ESM.pdf]

Additional appendix

Health gains and financial risk protection afforded by public financing of selected malaria interventions in Ethiopia: an extended cost-effectiveness analysis

By

Lelisa Fekadu Assebe, Xiaoxiao Jiang Kwete, Dan Wang, Lingrui Liu, Ole Frithjof Norheim, Abdulrahman Jbaily, Stéphane Verguet, Kjell Arne Johansson, Mieraf Taddesse Tolla

1. Introduction

This appendix describes the assumptions underlying the methodology used, and presents supplementary tables, figures, and sensitivity analyses used for the extended cost-effectiveness analysis (ECEA) of universal public finance (UPF) of selected malaria preventive and curative interventions. The methodology for the four malaria interventions is described under section 2 and builds on a previous ECEA of malaria vaccine in Zambia [1].

1.1. Description of model inputs and assumptions for all the interventions

The population at risk of malaria (about 60% of the total Ethiopian population) is the target population for long-lasting insecticide-treated bednets (LLIN) and indoor residual spraying (IRS); for artemisinin combination therapy (ACT), the target population is the estimated number of annual malaria cases of 2016. For malaria vaccine, the target population is the Ethiopian 2016 birth cohort (i.e. calculated as a product of crude birth rate by the size of the at-risk population) in at-risk areas followed over five years to capture the potential full impact of the vaccine. Each target population was evenly distributed across income quintiles for LLIN, IRS and ACT interventions. For the vaccine, quintile-specific total fertility rates were applied in order to differentiate the number of susceptible infants across income quintiles [2].

For each intervention, to distribute the prevalence of malaria for the at-risk population across income quintiles, we used the average malaria prevalence across socioeconomic groups with two diagnostic methods (microscopy and rapid diagnostic test) from the 2015 Malaria Indicator

Survey and the proportion of clinical malaria cases (i.e. 0.5%) from Ethiopia's Federal Ministry of Health (FMOH) malaria review report [3,4].

In order to calculate malaria prevalence by at-risk population per income quintile, we first estimated the relative risk of malaria prevalence by income quintile, and then multiplied it with the prevalence of malaria for the at-risk population. The distribution of malaria cases into outpatient and inpatient categories followed the share of malaria-related hospital admissions and was further disaggregated by income quintile with the distribution of malaria prevalence across income quintiles [5,6].

Case fatality ratios (CFR) for both outpatient and inpatient cases were extracted from the World Health Organization (WHO) 2015 and 2016 malaria reports, which were assumed to be similar across quintiles [7,8]. Then, for all the interventions (except vaccine), we distributed the baseline malaria-related deaths by income quintile through the product of outpatient and inpatient CFR by the number of outpatient and inpatient malaria cases, respectively.

Regarding malaria vaccine, at baseline, among the total malaria deaths, 48% of deaths would occur among under-five children [9]. The total number of malaria deaths was multiplied by this proportion in order to obtain the number of malaria deaths among under-five children [8,9]. Furthermore, malaria deaths were disaggregated by age group, as vaccine efficacy would wane with time since vaccination [8,9]. We used proxy measures (prevalence, treatment coverage, efficacy and child mortality) to distribute the malaria-related deaths by income quintile [10]. We estimated a relative risk ratio of dying from malaria between two income groups j and k as:

$$\frac{R_j}{R_k} \sim \frac{5q0_j \times (1 - aCOV_j Eff)}{5q0_k \times (1 - aCOV_k Eff)}, \quad (1)$$

where $5q0_j$ is under-five mortality in income quintile j, $aCOV_j$ is malaria treatment coverage in income group j as provided by EDHS 2016 [2], and Eff is treatment effectiveness (assumed constant across quintiles for simplicity) [11]. The risk index in equation (1) (i.e. R_k) is estimated as an average of three proxy measures: probability of being infected with malaria, malaria treatment seeking and a proxy for the relative probability of dying from childhood illness. This approach enables us to distribute the baseline child deaths due to malaria in each quintile. In addition, a Weibull decay function was used to take into account the waning of the vaccine over

the five-year time horizon: $E(t) = e_0\ exp^{\left(-ln(2)*\frac{(t-to)K}{L^K}\right)}$, where E_0 is initial efficacy against infection (91.1% following third dose), L is half-life protection, K is the decay shape, and $(t - t_0)$ is the time since vaccination [12]. The birth cohort would receive three vaccine doses over 6, 7.5 and 9 months, where vaccine would offer protection starting at age 9 months. UPF would yield a 10% incremental coverage across quintiles for all four interventions.

2. ECEA of malaria interventions

For the three preventive (LLIN, IRS and vaccine) and one curative (ACT) malaria intervention, we divide the population into five income groups j, and we denote y_j the average individual consumption expenditures per income quintile. $p_{in,j}$ denotes the proportion of inpatient malaria cases, and $p_{out,j}$ denotes the proportion of outpatient malaria cases in income quintile j ; and health care utilization is denoted u_j . $OOP_{in,j}$ are the OOP costs of inpatient visit for malaria, and $OOP_{out,j}$ are the OOP costs of outpatient visit for malaria among income group j ; $OOP_{total,j}$ is the total OOP costs in income quintile j. $C_{in,gov,j}$ and $C_{out,gov,j}$ are the government costs for inpatient and outpatient visit for malaria disease treatment in income group j. The intervention has an effectiveness *Eff*; the incremental coverage achieved by the program is Cov_j.

2.1. Estimation of health benefits (i.e. deaths averted)

The number of deaths averted by the intervention in income group j was expressed with a simple static model:

$$D_{av,j} = \left(Eff * Cov_j * D_j\right) \quad , \quad (2)$$

where D_j is the annual number of malaria-related deaths (among under-fives or among all age groups) in income quintile j before the program, and Cov_j is incremental coverage.

2.2. Consequences for household expenditures

We estimated the private expenditures averted in each income quintile j for both preventive interventions (vaccine, LLIN, IRS) and curative interventions (ACT) potentially rolled out in Ethiopia. For preventive interventions, the private expenditures averted by public finance in each income quintile j would be computed as:

$$\mathrm{PE}_{av,j} = Eff * Cov_j * u_j * [\, p_{in,j} * OOP_{in,j} + p_{out,j} * OOP_{out,j}] * n_j \quad , \quad (3)$$

where n_j is the annual number of malaria cases (among under-fives or among all age groups) in income quintile j before the program.

For curative interventions (i.e. ACT), the private expenditures averted by publicly finance in each income quintile j would be computed as:

$$\mathrm{PE}_{av,j} = u_j * [p_{in,j} * OOP_{in,j} + p_{out,j} * OOP_{out,j}] * n_j \quad , \quad (4)$$

where n_j is the annual number of malaria cases (among under-fives or among all age groups) in income quintile j before the program, as before-the-program out-of-pocket (OOP) costs are removed by public finance.

2.3. Estimation of financial risk protection benefits

A case of catastrophic health expenditure (CHE) before intervention (CHE_0) is counted when OOP spending for malaria care ($OOP_{in,j}$ or $OOP_{out,j}$ above) is higher than a specified threshold (Th =10%) defined in comparison with consumption expenditures per quintile (i.e. y_j). Then, CHE_0 among those who utilized care occur when $OOP_{in,j}$ or $OOP_{out,j}$ > Th* y_j.

For preventive interventions (vaccine, IRS, LLIN), the introduction of public finance would avert the following number of CHE cases per income quintile j:

$$CHE_{av,j} = Cov_j * Eff * CHE_0 \quad . \quad (5)$$

For curative interventions (ACT), the introduction of public finance would avert the following number of CHE cases per income quintile: $Cov_j * CHE_0$.

Cases of CHE were estimated using either a threshold of annual income or a capacity to pay approach (Table S1). For capacity to pay, we extracted the proportion of food expenditure (FE_j) per income quintile j. Then, we calculated the absolute value of subsistence expenditure (SE_j) in quintile j as $SE_j = (1- FE_j)*y_j$. Capacity to pay was calculated as $y_j - SE_j$ [13,14].

2.4. Quantification of the total costs of the program

From the government perspective, the total costs incurred for the vaccine program are, per income quintile:

$$TC_{Vac,j} = Cov_j * C_{vac} * Popj \quad , \quad (6)$$

where C_{vac} stands for both the costs of the vaccine (3 doses) and program implementation, Cov_j is vaccine coverage per quintile, and $Popj$ is the target population per quintile. The healthcare costs of malaria treatment averted by vaccine for the government (per quintile j) are:

$$TC_{HC,j} = Eff * Cov_j * u_j * [(p_{in,j} * C_{in,gov,j} + p_{out,j} * C_{out,gov,j})] * n_j \quad , \qquad (7)$$

where n_j is the annual number of malaria cases (among under-fives or among all age groups) in income group j before program. Hence, from the government perspective, the net incremental costs incurred are:

$$TC = TC_{Vac,j} - TC_{HC,prev,j} \quad . \qquad (8)$$

From the government perspective, the total incremental costs incurred for LLIN/IRS program are, per income quintile:

$$TC_{prev,j} = Cov_j * c_{gov} * Popj \quad , \qquad (9)$$

where c_{gov} is the unit costs of LLIN/IRS intervention, $Popj$ is the target population per quintile, and Cov_j is incremental coverage (10%). The total LLIN cost is adjusted by one half, corresponding to one net per two people within a household.

The healthcare costs of malaria treatment averted by LLIN/IRS intervention for the government (per quintile j) are:

$$TC_{HC,prev,j} = Eff * Cov_j * u_j * [(p_{in,j} * C_{in,gov,j} + p_{out,j} * C_{out,gov,j})] * n_j \quad , \qquad (10)$$

where n_j is the annual number of malaria cases (among under-fives or among all age groups) in income quintile j before program, and Cov_j is incremental coverage (10%). Hence, from the government perspective, the net incremental costs incurred are:

$$TC = TC_{prev,j} - TC_{HC,prev,j} \quad . \qquad (10)$$

From the government perspective, for ACT, the incremental government expenditure per quintile are given by:

$$TC_{cure,j} = Cov_j * n_j * \left(p_{in,j} * C_{in,gov,j} + p_{out,j} * C_{out,gov,j}\right) + \left(p_{in,j} * OOP_{in,j} + p_{out,j} * OOP_{out,j}\right) * u_j * n_j \quad , \qquad (11)$$

where n_j is the annual number of malaria cases (among under-fives or among all age groups) in income quintile j before program; u_j is healthcare utilization before program, and Cov_j is the incremental coverage (10%).

3. Additional tables and figures

Table S1. Cases of catastrophic health expenditure averted, for public finance of malaria interventions after a 10% increase in coverage, in Ethiopia.

**Interventions**	**Cases of catastrophic health expenditures averted**	**Cases of catastrophic health expenditures averted (40% capacity to pay)**
Artemisinin-based combination	440	182
Long-lasting insecticide-treated bednets	220	91
Indoor residual spray	125	52
Malaria vaccine	18	9

Annual parasite incidence (API) corresponds to the total number of positive confirmed cases per 1000 population per year [15]. The API level for a specific geographic area is used to classify the districts into control (i.e. API ≥ 10), optimization (i.e. 5 < API < 10), pre-elimination and elimination phases (i.e. 0 < API < 5). As shown in Table S2, the health impact of all malaria interventions in the control phase would be substantial, however in other phases selected interventions would yield more benefit.

Table S2: Extended cost-effectiveness analysis results for each intervention per malaria transmission intensity: deaths averted, out-of-pocket (OOP) expenditures averted, and cases of catastrophic health expenditures (CHE) averted.

**Intervention**	**Outcome**	**0<API< 5 (Pre-elimination /Elimination)**	**5 ≥ API < 10 (Optimization phase)**	**API ≥ 10 (Control phase)**
LLINs	Deaths averted	4	14	102
	OOP expenditures averted	3,696	16,262	106,213
	Cases of CHE averted	7	20	179
IRS	Deaths averted	2	8	58
	OOP expenditures averted	2,107	9,269	60,541
	Cases of CHE averted	4	11	102
Malaria vaccine	Deaths averted	0	1	10
	OOP expenditures averted	300	1,321	8,627
	Cases of CHE averted	2	10	88
ACT	Deaths averted	8	27	194
	OOP expenditures averted	73,913	325,232	2,124,255
	Cases of CHE averted	13	38	340

Figure S1. Distribution of deaths averted and financial risk protection afforded per US$1 million spent in each income quintile for malaria interventions (Q1 is poorest and Q5 is richest) in Ethiopia.

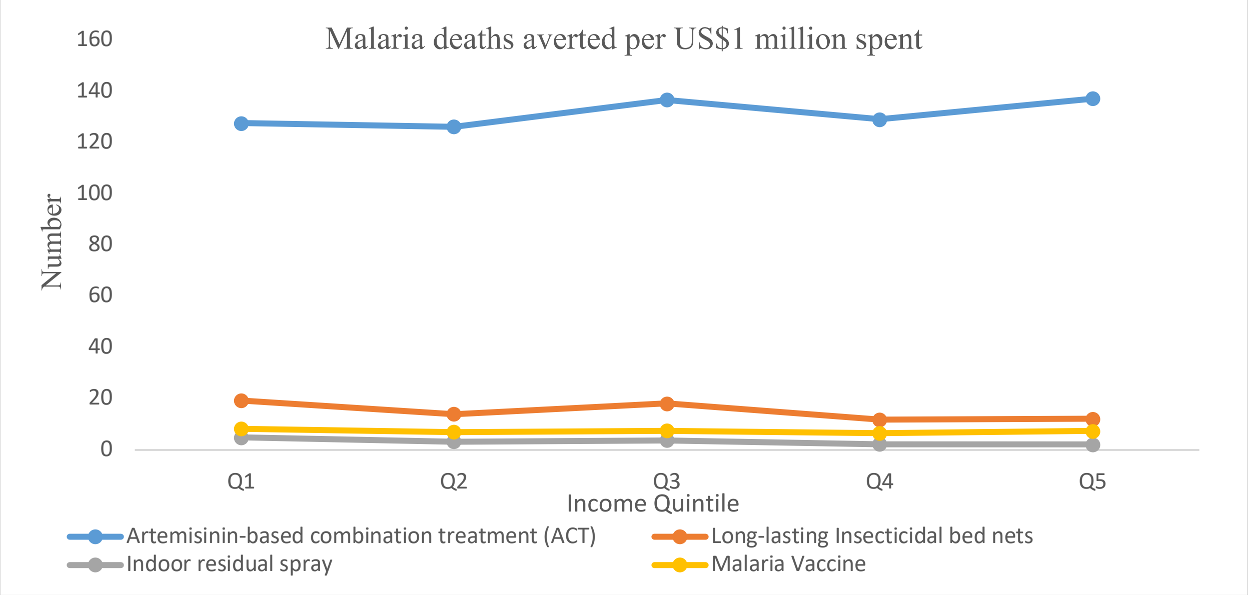

Figure S2. Distribution of financial risk protection afforded per US$1 million government expenditures for each of malaria intervention per income quintile (Q1 is poorest and Q5 is richest) in Ethiopia.

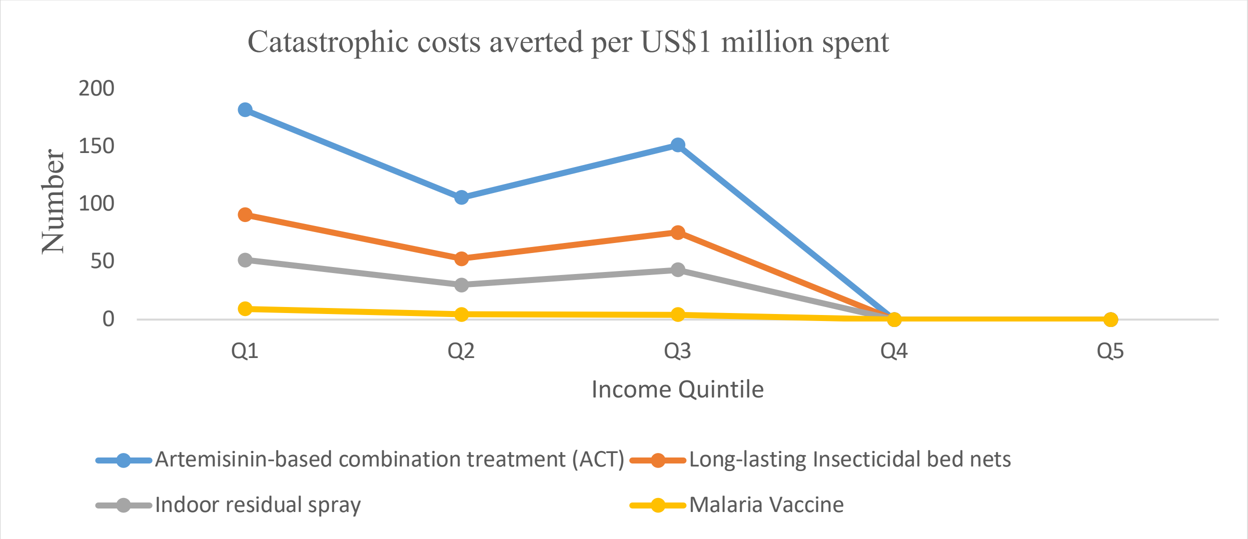

Figure S3. Private expenditure averted (in USD) and malaria deaths averted, per $1 million net government expenditures, per income quintile, by malaria preventive intervention in Ethiopia.

Q1 = Poorest; Q2 = Poorer; Q3 = Middle; Q4 = Richer; Q5 = Richest.

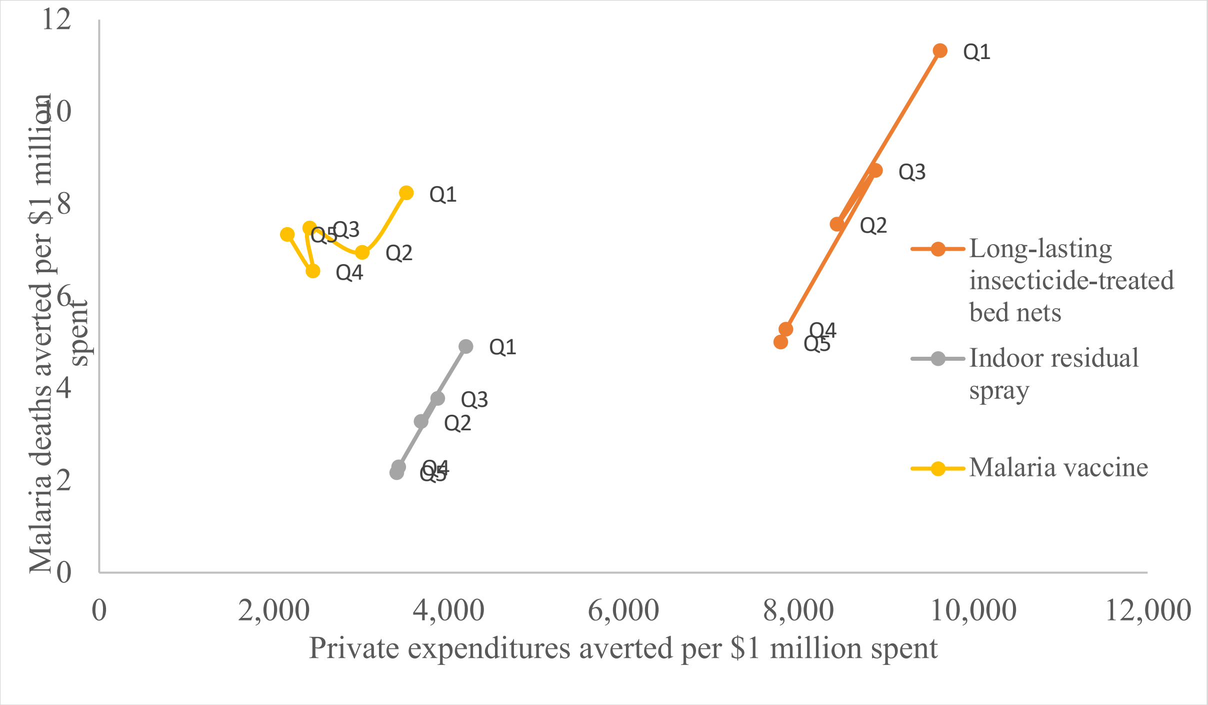

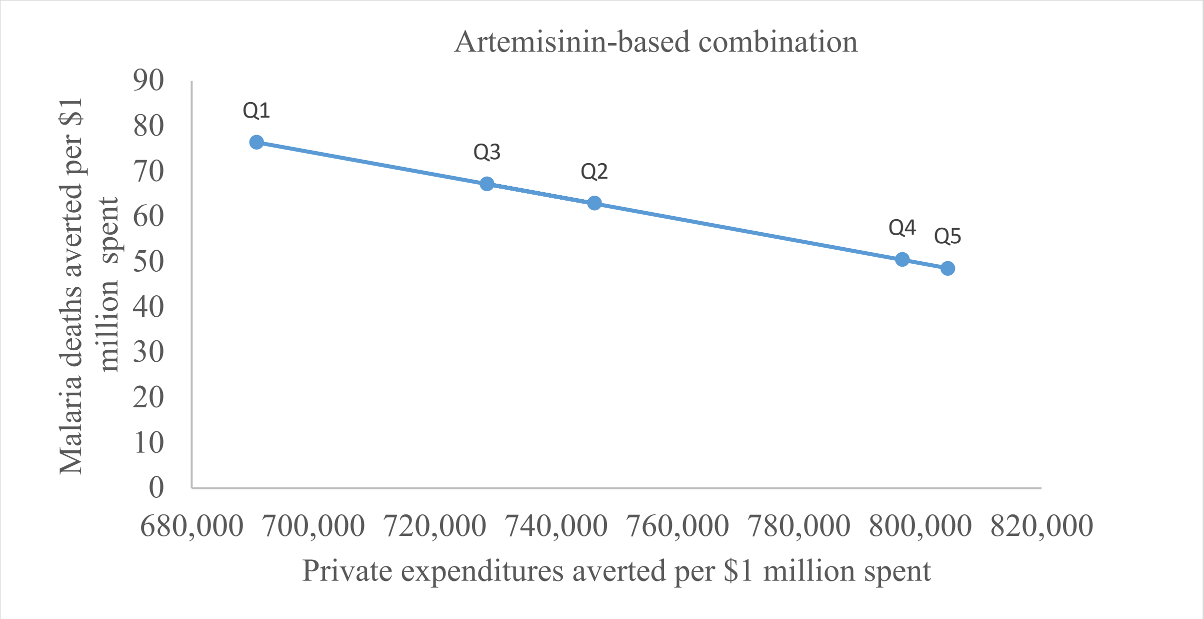

4. **Sensitivity analyses**

Table S3: Sensitivity analysis of deaths averted and cases of catastrophic health expenditures (CHE) when IRS model input parameters were varied across income quintiles (Q1 = poorest; Q5 = richest), (low to high shows when model input parameters are decreased/increased, respectively).

**Sensitivity analysis IRS**	**Q1**		**Q2**		**Q3**		**Q4**		**Q5**	
	**Low**	**High**	**Low**	**High**	**Low**	**High**	**Low**	**High**	**Low**	**High**
**Prevalence of malaria**										
Deaths averted	26	39	17	26	20	30	12	18	12	17
Private expenditures averted ($1,000s)	22	33	19	29	21	31	18	27	18	27
CHE cases averted	42	62	24	36	35	52	0	0	0	0
**Malaria case fatality ratio**										
Death averted	26	38	17	26	20	29	12	18	11	17
Private expenditures averted ($1,000s)	28	28	24	24	25	25	22	22	22	22
CHE cases averted	52	52	30	30	43	43	0	0	0	0
**Health care use**										
Death averted	32	32	21	21	25	25	15	15	14	14
Private expenditures averted ($1,000s)	22	33	19	29	20	31	18	27	18	27
CHE cases averted	42	63	24	36	35	52	0	0	0	0
**Probability of inpatient visit**										
Death averted	32	32	21	22	25	25	15	15	14	14
Private expenditures averted ($1,000s)	33	34	28	29	30	31	27	27	27	27
CHE cases averted	50	75	29	43	41	62	0	0	0	0
**Efficacy**										
Death averted	26	38	17	26	20	30	12	18	11	17
Private expenditures averted ($1,000s)	22	33	19	29	20	31	18	27	18	27
CHE cases averted	42	62	24	36	35	52	0	0	0	0
**Cost inputs /IRS**										
Government costs for the policy ($1,000s)	5216	7838	5220	7842	5214	7836	5221	7843	5218	7840
**OOP outpatient /IRS**										
Death averted	32	32	21	21	25	25	15	15	14	14
Private expenditures averted ($1,000s)	23	32	20	29	21	30	19	27	18	26
CHE cases averted	52	52	30	30	43	43	0	0	0	0

Table S4: Sensitivity analysis of deaths averted and cases of catastrophic health expenditures (CHE) averted when malaria vaccine model input parameters were varied across income quintiles (Q1 = poorest; Q5 = richest), (low to high shows when the model input parameters are decreased or increased, respectively).

Sensitivity analysis vaccine	Q1		Q2		Q3		Q4		Q5	
	Low	High	Low	High	Low	High	Low	High	Low	High
**Prevalence of malaria**										
Deaths averted	9	11	7	8	6	8	5	6	3	4
Private expenditures averted	3 900	5 850	2 930	4 390	2 040	3 070	1 820	2 730	972	1 460
CHE cases averted	7	11	4	5	3	5	0	0	0	0
**Malaria case fatality ratio**										
Deaths averted	9	11	7	8	6	8	5	6	3	4
Private expenditures averted	4 880	4 880	3 660	3 660	2 560	2 560	2 280	2 280	1 210	1 210
CHE cases averted	9	9	5	5	4	4	0	0	0	0
**Health care use**										
Deaths averted	11	11	8	8	8	8	6	6	4	4
Private expenditures averted	3 940	5 900	2 890	4 330	2 050	3 070	1 810	2 710	981	1 470
CHE cases averted	5	14	2	7	2	7	0	0	0	0
**Probability of inpatient visit**										
Deaths averted	11	11	8	8	8	8	6	6	4	4
Private expenditures averted	4 770	4 990	3 610	3 710	2 500	2 610	2 250	2 300	1 200	1 230
CHE cases averted	7	11	4	5	3	5	0	0	0	0
**Efficacy**										
Death averted	9	14	7	10	6	10	5	7	3	5
Private expenditures averted	3 900	5 850	2 930	4 390	2 050	3 070	1 820	2 730	970	1 460
CHE cases averted	7	11	4	5	3	5	0	0	0	0
**Cost inputs /vaccine**										
Government costs for the policy	1 108 670	1 665 610	970 860	1 458 170	849 670	1 276 070	745 928	1 120 118	451 020	677 275
**OOP outpatient /vaccine**										
Deaths averted	11	11	8	8	8	8	6	6	4	4
Private expenditures averted	4 020	5 730	2 990	4 330	2 100	3 010	1 850	2 710	990	1 440
CHE cases averted	9	9	5	5	4	4	0	0	0	0

Table S5: Sensitivity analysis of death averted and cases of catastrophic health expenditures (CHE) averted when ACT model input parameters were varied across income quintiles (Q1 = poorest; Q5 = richest), (low to high shows when the model input parameters are decreased or increased, respectively).

**Sensitivity analysis ACT**	**Q1**		**Q2**		**Q3**		**Q4**		**Q5**	
	**Low**	**High**	**Low**	**High**	**Low**	**High**	**Low**	**High**	**Low**	**High**
**Prevalence of malaria**										
Deaths averted	86	128	57	86	67	100	40	60	38	58
Private expenditures averted ($1,000s)	774	1161	678	10167	722	10823	6367	955	6367	954
Cases of CHE averted	146	219	85	127	123	184	0	0	0	0
**Malaria case fatality ratio**										
Deaths averted	86	127	58	85	66	98	40	60	38	57
Private expenditures averted ($1,000s)	966	966	847	847	892	892	789	789	783	783
Cases CHE averted	182	182	106	106	152	152	0	0	0	0
**Health care use**										
Deaths averted	107	107	71	71	82	82	50	50	47	47
Private expenditures averted ($1,000s)	779	1169	669	1004	714	1070	627	940	632	949
Cases CHE averted	147	220	84	125	121	182	0	0	0	0
**Probability of inpatient visit**										
Deaths averted	106	108	71	72	82	83	50	50	47	47
Private expenditures averted ($1,000s)	945	988	835	860	874	910	78	798	773	792
Cases of CHE averted	146	219	85	127	121	182	0	0	0	0
**Efficacy**										
Deaths averted	86	113	57	75	66	87	40	53	38	50
Private expenditures averted ($1,000s)	966	966	847	847	892	892	789	789	783	783
Cases of CHE averted	182	182	106	106	152	152	0	0	0	0
**Cost inputs /ACT/**										
Government costs for the treatment (outpatient cost varied) ($1,000s)	1316	1479	1079	1189	1160	1286	950	1028	935	1009
Government costs for the treatment (inpatient cost varied) ($1,000s)	1394	144	1133	1138	1221	1227	989	991	92	974
**OOP outpatient /ACT/**										
Deaths averted	107	107	71	71	82	82	50	50	47	47
Private expenditures averted ($1,000s)	797	1135	692	1003	734	1050	641	937	636	929
Cases of CHE averted	182	182	106	106	152	152	0	0	0	0

Table S6: Sensitivity analysis of deaths averted when all deaths occurring in the general population is assumed to occur in population at risk (i.e. high case scenario, 5,000 and base case scenario, 3,767) at baseline.

	Intervention	Scenario	Average	Q1	Q2	Q3	Q4	Q5
**Sensitivity analysis**	ACT	Base case	358	107	71	82	50	47
		High case	475	141	95	109	67	63
	LLIN	Base case	188	56	38	43	26	25
		High case	250	74	50	57	35	33
	IRS	Base case	107	32	21	25	15	14
		High case	143	42	28	33	20	19
	Vaccine	Base case	38	11	8	8	6	4
		High case	51	15	11	11	8	6

As shown in the above table if all malaria related deaths were attributed to population at risk, the death averted proportion would increase by approximately 42% for all interventions.

References

1. Liu L, Portnoy A, True Z, Fink G, Verguet S. The health and financial benefits for households from averting malaria with RTS, S/AS01 vaccine in Zambia: an extended cost-effectiveness analysis. Disease Control Priorities in Developing Countries,3rd Edition,Working Paper No. 26. Seattle: University of Washington, 2018.
2. Central Statistical Agency, Federal Democratic Republic of Ethiopia. Ethiopia Demographic and Health Survey. Addis Ababa, Ethiopia; Rockville, Maryland, USA: ICF International, central Statistical Agency; 2016.
3. Ethiopian Public Health Institute. The 2016 Ethiopia National Malaria Indicator Survey (EMIS). Addis Ababa, Ethiopia: Ethiopian Public Health Institute; 2016.
4. Ethiopia Federal Ministry of Health. Ethiopian External Malaria Program midterm review. Addis Ababa, Ethiopia: Federal Ministry of Health; 2017.
5. Jima D, Wondabeku M, Alemu A, Teferra A, Awel N, Deressa W, et al. Analysis of malaria surveillance data in Ethiopia: what can be learned from the Integrated Disease Surveillance and Response System? Malaria Journal 2012; 11(1):330.
6. President's Malaria Initiative (PMI). 2017 Ethiopia Malaria Operational Plan. 2017.
7. World Health Organization. World malaria report 2015. Geneva: World Health Organization; 2015.
8. World Health Organization. World malaria report 2017. Geneva: World Health Organization; 2017.
9. Institute for Health Metrics and Evaluation. Global Burden of Disease (GBD) 2016 data. Retrieved from: https://vizhub.healthdata.org/gbd-compare/ (accessed May 10, 2018).
10. Rheingans RD, Atherly D, Anderson J. Distributional impact of rotavirus vaccination in 25 Gavi countries: estimating disparitires in benefits and cost-effectiveness. Vaccine 2012; 30:A15-A23.
11. Penny MA, Ross PP, Smith TA. The time-course of protection of the RTS,S vaccine against malaria infections and clinical disease. Malaria Journal 2015; 14:437.
12. Daivadanam M, Thankappan KR, Sarma PS, Harikrishnan S. Catastrophic health expenditure and coping strategies associated with acute coronary syndrome in Kerala, India. Indian J Med Res 2012; 136(4):585-92.
13. National Planning commission Ethiopia. Ethiopia’s Progress Towards Eradicating Poverty፡ An Interim Report on 2015/16 Poverty Analysis Study. September 2017.
14. Federal Ministry of Health. National Malaria Strategic Plan 2017–2020. April 2017.
